# Patient counseling for pulmonary embolism requires an individualized approach

**DOI:** 10.1016/j.rpth.2024.102407

**Published:** 2024-04-12

**Authors:** Carolyn G. Chatterton, Lina A. Fouad, Jeffrey A. Kline

**Affiliations:** 1 Department of Obstetrics and Gynecology, Division of Maternal Fetal Medicine, Wayne State University School of Medicine, Detroit, Michigan, USA; 2 Department of Emergency Medicine, Wayne State University School of Medicine, Detroit, Michigan, USA

Pulmonary embolism (PE) remains a major source of morbidity and mortality in pregnancy [1]. Classically, PE in pregnancy is diagnosed via radiographic evaluation with either a computed tomography pulmonary angiography (CTPA) or a ventilation/perfusion scan. Both of these modalities effectively rule out PE and have similar safety profiles [2]. Society recommendations from the United States, the United Kingdom, and Canada support the use of CTPA and ventilation/perfusion scans in pregnancy for the purposes of evaluating for PE when indicated as the benefits of prompt diagnosis outweigh the risk of radiation [[3], [4], [5], [6]]. However, the decision to order these tests in pregnancy is inherently more complex than in the nonpregnant patient. Normal physiological changes of pregnancy, including dyspnea, tachycardia, and lower extremity swelling, can mimic the signs and symptoms of PE. Pregnancy also increases the baseline risk for developing venous thromboembolism (VTE) or PE, and expedient diagnosis and treatment may be life-saving. While the dose of radiation is considered safe for the mother and fetus, it is not without risk [7]. Clinicians necessarily have a lower threshold to evaluate for PE in the pregnant patient than in the nonpregnant patient and must balance the nuanced risks and benefits of imaging recommendations for both the mother and the fetus, despite there being no randomized control trials to guide clinical decision-making. It is crucial that clinicians evaluating pregnant patients with a potential PE understand the risks and benefits of the diagnostic modalities employed.

In this issue of *RPTH*, Ouellet et al. [8] provide data about what is important from the pregnant patient’s perspective. The authors point out that pretest counseling for pregnant patients undergoing diagnostic evaluation for PE is, at best, subpar. As part of a larger project to develop a tool for patient education regarding PE in pregnancy, Ouellet et al. [8] interviewed 20 pregnant patients about their counseling needs. The authors concluded that patients wished to be informed about the maternal and fetal risks of PE and radiation associated with imaging and that a written tool may be helpful in delivering this information. Effective pretest counseling by the clinician relies on a robust understanding of an individual patient’s pretest probability. The Wells criteria are commonly used to determine pretest probability of PE in nonpregnant patients. However, this pretest calculator has not been validated in the pregnant population [9]. There has been a recent push to incorporate a more nuanced approach to pretest probability in pregnant patients with possible PE [10].

Both the CT-PE Pregnancy Group Study and the Artemis Study define a framework for pretest probability, neither of which was addressed by the patient interviews in the study by Ouellet et al. [8,11,12]. The CT-PE Pregnancy Group Study assessed pretest probability for pregnant patients in Europe with possible PE using a combination of the revised Geneva score, which has not been validated in pregnancy, maternal serum D-dimer, and bilateral leg compression ultrasonography. CTPA was avoided in 11.6% of the 395 included patients [12].

In the Artemis Study, the authors prospectively evaluated the use of a pregnancy-modified algorithm incorporating the YEARS criteria (clinical signs of deep venous thrombosis, hemoptysis, and PE as the most likely diagnosis) in conjunction with maternal serum D-dimer in 498 patients referred for suspected PE in Europe. Using their algorithm, CTPA was avoided in 39% of all pregnant patients and in 65%, 46%, and 32% of patients in the first, second, and third trimesters, respectively. The decreasing specificity of the YEARS algorithm is likely due to physiological increase in D-dimer that occurs as pregnancy progresses [11]. When the pregnancy-modified YEARS algorithm was retrospectively applied to the patient CT-PE Pregnancy Study population, 77 patients (21%) would have safely avoided CTPA [13]. The pregnancy-modified YEARS algorithm has since been evaluated in a US-based patient population, and the authors found that in their retrospective cohort of 77 patients, 34.3% of patients would have appropriately avoided radiation [14].

Since the publication of the 2018 CT-PE Pregnancy Study and the 2019 Artemis Study, professional society statements about the diagnosis of PE in pregnancy have not yet been updated to this algorithm [[3], [4], [5], [6],^10^]. Nonetheless, decision algorithms that incorporate the use of D-dimer and other clinical signs and symptoms allow clinicians to effectively stratify risk, and patients should be informed of this benefit. Despite no change in published society guidelines, the Artemis Study, in particular, has impacted clinical practice.

The authors cite the general incidence of PE in pregnancy in Canada as approximately 1 to 2 per 1000 pregnancies. According to the original citation by Liu et al. [15], this is actually the incidence of deep venous thrombosis in both the pregnancy and the postpartum periods. The incidence of PE in the antepartum period, according to Liu et al. [15], is 0.05%. A more recent 2015 meta-analysis that includes this reference, in addition to 26 other studies, found significant heterogenicity in the way incidence of PE is reported but reported that the majority of pregnancy-associated VTE events (57.5%) occur postpartum when the fetus is no longer a factor in the clinical decision-making for radiographic evaluation. During pregnancy, the authors found that the majority of VTE events occur in the third trimester (55.95%) when the fetus is outside the period of significant organogenesis [16]. It is important for counseling clinicians to consider the differences in VTE incidence and radiation risk according to period of gestation.

Information about clinical decision-making for radiography in pregnancy during a possible PE is important to relay to the patient in context of their individual clinical presentation and in a way that they may understand. Ouellet et al. [8] employed a qualitative approach for data collection and analysis, and there is an emphasis on the thematic trends identified among patients. The authors recruited patients who presented for routine obstetric care and had no signs or symptoms of PE. Accordingly, this patient sample may lack the feelings of urgency and acute emotional concern for clinical resolution that would often be present in the emergency care setting.

In this study, 20% of the participants had a history of VTE. The similarities and differences between the groups of patients with a history of VTE and those without were not discussed. It would have been interesting to compare these responses as patients with a history would be able to speak from their personal experience. Future work should interview subjects with a history of PE in pregnancy requiring imaging or at the time of consent for radiographic PE evaluation. Furthermore, because the majority of patients in this study have history of other comorbidities, there may be an increased baseline level of medical literacy or interest in improving their own medical education. Indeed, 70% of the participants had an identified comorbidity, although the details of what they were and what their impact on responses were remained lacking. For example, inherited thrombophilia and advanced maternal age are both risk factors for VTE in pregnancy, but these conditions each pose differing levels of risk. It would be important for the reader to understand which comorbidities are represented to further qualify the types of answers that were given and to better understand any subgroups that may emerge among the patient population.

The elaboration on the participants’ differing experiences with PE and the health care system provides important insight into the patient experience. Many participants had limited knowledge of a PE, how it is diagnosed, associated maternal and fetal risks, and approach to treatment. This inherent knowledge gap makes it difficult for subjects to provide meaningful responses that can shape their counseling. This is evidenced by the somewhat obvious conclusions of the study that 1) patients wish to know maternal and fetal risks of diagnostic imaging, and 2) patients may benefit from a written tool to read before deciding. Many hospital systems require patients to read and sign a consent form informing them of the risk of radiation exposure in pregnancy. However, it is unknown whether a written tool provided at the time of consent for radiation will provide significant improvement in factual knowledge or patient anxiety. Future studies may address this aspect of patient counseling.

This work highlights the need for improved pretest counseling when a pregnant patient is being evaluated for possible PE. Comprehensive and individualized counseling requires the provider to explain to the patient using evidence, conveyed in simple language, about her pretest probability of a PE as well as the risks of radiation to both mother and child and unknowns such as the effect of radiocontrast on fetal thyroid function. Further research is needed to understand if the patient’s need for information changes in an urgent or emergent setting and if subsets of patients, based on their history or other demographics, may benefit from a different counseling approach.
